# Deubiquitination of FBP1 by USP7 blocks FBP1–DNMT1 interaction and decreases the sensitivity of pancreatic cancer cells to PARP inhibitors

**DOI:** 10.1002/1878-0261.13149

**Published:** 2021-12-17

**Authors:** Xiang Cheng, Bin Zhang, Feng Guo, Heshui Wu, Xin Jin

**Affiliations:** ^1^ Department of Urology The Second Xiangya Hospital Central South University Changsha China; ^2^ Cancer center Union Hospital Tongji Medical College Huazhong University of Science and Technology Wuhan China; ^3^ Department of Pancreatic Surgery Union Hospital Tongji Medical College Huazhong University of Science and Technology Wuhan China; ^4^ Uro‐Oncology Institute of Central South University Changsha China

**Keywords:** DNMT1, FBP1, pancreatic cancer, PARP inhibitors, USP7

## Abstract

Poly[ADP‐ribose] polymerase (PARP) inhibitors can block DNA single‐strand damage repair and subsequently increase double‐stranded breaks (DSBs) by reducing the activity of the PARP1 protease and by preventing the PARP1 protein from dissociating from chromatin. Tumors with the BRCA mutation are particularly sensitive to PARP inhibitors. So far, PARP inhibitors (Olaparib) have been used to treat pancreatic cancer patients with BRCA mutation. However, these patients are prone to PARP inhibitor resistance. Our previous studies suggest that fructose‐1,6‐bisphosphatase 1 (FBP1) is responsible for the sensitivity to various anticancer agents, such as gemcitabine or mitogen‐activated protein kinase kinase (MEK) inhibitors. In this study, we demonstrate that FBP1 regulates the sensitivity to PARP inhibitors in pancreatic cancer. Then, we showed that nuclear FBP1 is responsible for this process by interacting with DNA (cytosine‐5)‐methyltransferase 1 (DNMT1) and trapping PARP1 in chromatin. Moreover, we revealed that ubiquitin carboxyl‐terminal hydrolase 7 (USP7) binds to and induces the deubiquitination of FBP1, which prevented FBP1 from translocating to the nucleus. Finally, we demonstrated that USP7 inhibitors enhanced the antitumor effect of PARP inhibitors in an FBP1‐dependent manner. Collectively, our results identify a novel USP7–FBP1–DNMT1 signaling axis in pancreatic cancer, which might indicate that USP7 inhibitors and PARP inhibitors might have more powerful antitumor effects than PARP inhibitors alone in pancreatic cancer patients.

## Graphical abstract

In this study, we aimed to understand how FBP1 regulates sensitivity to PARP inhibitors in pancreatic cancer. We found that FBP1 localized in the nucleus interacted with DNMT1 and trapped PARP1 to the chromatin. Furthermore, FBP1 de‐ubiquitination induced by binding to ubiquitin carboxyl‐terminal hydrolase 7 (USP7) inhibited the ability of FBP1 to translocate to the nucleus. We therefore suggest that USP7 inhibitors in combination with PARP inhibitors might manifest more powerful antitumor effects than PARP inhibitors alone in pancreatic cancer cells.

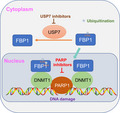

**Abbreviations**
ANOVA: analysis of variance
Co‐IP: coimmunoprecipitation
DMEM: Dulbecco’s modified Eagle’s medium
DNMT1: DNA (cytosine‐5)‐methyltransferase 1
DSB: double‐stranded breaks
FBP1: fructose‐1,6‐bisphosphatase 1
GST: glutathione S‐transferase
HR: homologous recombination
IC50: the half‐maximal inhibitory concentration
MEK: mitogen‐activated protein kinase kinase
PARP: Poly[ADP‐ribose] polymerase
shRNAs: short hairpin RNAs
USP7: ubiquitin carboxyl‐terminal hydrolase 7

## 1 Introduction

Pancreatic cancer is a fatal tumor of the digestive tract that is highly malignant. Complete resection of the tumor is currently the main effective treatment for pancreatic cancer [1]. However, two‐thirds of patients with pancreatic cancer are diagnosed at the late stage and have lost the opportunity for radical resection. For these patients, chemotherapy or chemoradiotherapy slightly prolonged their survival time [2]. Traditional chemotherapy drugs for pancreatic cancer mainly include gemcitabine, albumin paclitaxel, or fluorouracil [2]. Clinical evidence indicated that patients with pancreatic cancer are highly resistant to these chemotherapeutic agents [2]. Therefore, identification of new treatment strategies for pancreatic cancer is urgently needed.

The heterogeneity of pancreatic cancer suggests that the mutation or dysfunction of multiple genes is closely associated with its tumorigenesis and drug resistance of pancreatic cancer [3]. Currently, small molecules targeting genes with a single mutation or abnormally activated pathways cannot significantly prolong the survival time of patients with pancreatic cancer [4, 5]. Therefore, a combination of two or more antitumor molecules targeting different genes or signaling pathways is expected to improve the prognosis of patients with pancreatic cancer.

DNA damage activates poly[ADP‐ribose] polymerase (PARP), which can recognize and bind to DNA single‐strand breaks and catalyze the poly[ADP–ribosylation] of receptor proteins to repair damaged DNA [6]. This process can reduce the occurrence of DNA double‐stranded breaks (DSBs) [6]. PARP inhibitors can block DNA single‐strand damage repair and subsequently increase DSBs by reducing the activity of the PARP1 protease and preventing the PARP1 protein from dissociating from chromatin [6]. Studies have shown that in many tumors (such as ovarian cancer and breast cancer), because of the BRCA mutation, the DSB repair process is impaired. These tumors are particularly sensitive to PARP inhibitors [7, 8]. At present, various PARP inhibitors have been used in the treatment of cancer patients with BRCA mutations in the clinic [9, 10]. However, only a small portion of patients with pancreatic cancer carry BRCA mutations [11, 12]. Moreover, patients with pancreatic cancer are prone to PARP inhibitor resistance [11, 12]. Thus, expanding the scope of application of PARP inhibitors and reversing drug resistance in pancreatic cancer are the key issues to be solved.

Our previous studies suggest that fructose‐1,6‐bisphosphatase 1 (FBP1) is responsible for the sensitivity to various anticancer agents, such as gemcitabine or mitogen‐activated protein kinase kinase (MEK) inhibitors [13, 14, 15]. Here, we showed that FBP1 translocated into the nucleus and bound to DNA (cytosine‐5)‐methyltransferase 1 (DNMT1) to modulate the sensitivity of pancreatic cancer cells to PARP inhibitors. Moreover, the subcellular localization of FBP1 was mediated by ubiquitin carboxyl‐terminal hydrolase 7 (USP7). Finally, we demonstrated that USP7 inhibitors enhanced the antitumor effect of PAPR inhibitors in an FBP1‐dependent manner. Therefore, our results identify a novel therapeutic strategy for pancreatic cancer.

## 2 Materials and methods

### 2.1 Cell lines, cell culture, and cell transfection

The pancreatic cancer cell lines MIAPaCa‐2 were purchased from Type Culture Collection of the Chinese Academy of Sciences (Shanghai, China). The Capan‐1 cells were obtained from YuchiCell Biological Technology (Shanghai, China). All of the cells had the STR authentication. The MIA PaCa‐2 cells are a BRCA1/2 wild‐type model and the Capan‐1 cells are a BRCA2 mutant type model for the subsequent study [16]. MIA PaCa‐2 cells were cultured in Dulbecco’s modified Eagle’s medium (DMEM; Thermo Fisher Scientific, Waltham, MA, USA) medium supplemented with 10% fetal bovine serum (HyClone, Logan, UT, USA) at 37 °C in a 5% CO2 incubator. Capan‐1 cells were cultured in IMDM (Thermo Fisher Scientific) medium supplemented with 20% fetal bovine serum (HyClone) at 37 °C in a 5% CO2 incubator. For cell transfection and infection, the detail procedure was described previously [17]. The specific sequences of shFBP1 are provided in the Table S1.

### 2.2 Cell proliferation assay

The MTS kit (ab197010, Abcam, Chicago, IL, USA) and CCK‐8 kit (C0037, Beyotime Biotechnology, Shanghai, China) were used the cell proliferation assay. The detail of procedure was described previously [17].

### 2.3 Mice study

CAnN. Cg‐Foxn1nu/Crl for BALB/c nude mice (4 weeks old, 18–20 g) was obtained from Vitalriver (Beijing, China). All mice were housed in standard conditions with a 12‐h light/dark and access to food and water *ad libitum*. Capan‐1 cells (5 × 10^6^) with different treatments were dispersed in 100 μL PBS and inoculated subcutaneously into the left dorsal flank of nude mice. The detail of procedure was described previously [18]. Experimental procedures were performed according to guidelines set forth by the Chinese National Institutes of Health and approved by the Ethical Committee on Animal Experiments of the Huazhong University of Science and Technology in Wuhan, China (Animal license number: 2502).

### 2.4 Reagent and antibodies

Gemcitabine (#S1714), MK2206 (#S1078), Trametinib (#S2673), Olaparib (#S1060), P005091 (#S7132), Decitabine (# S1200) were purchased from Selleck Chemicals (Shanghai, China). FBP1 antibody (# ab109732, working dilution 1 : 1000), GAPDH antibody (# ab8245, working dilution 1 : 5000), DNMT1 antibody (# ab92314, working dilution 1 : 1000), and PARP1 antibody (# ab32138, working dilution 1 : 1000) were purchased from Abcam. USP7 antibody (# 66514‐1‐Ig, working dilution 1 : 1000) was obtained from Proteintech (Wuhan, China). The KOD‐plus‐mutagenesis kit (#SMK101, TOYOBO LIFE SCIENCE, Osaka, Japan) was used to generate FBP1 mutants mentioned in the results.

### 2.5 Western blot and coimmunoprecipitation assay

Cells were lysed with the RIPA buffer (P0013B, Beyotime Biotechnology) on ice for 30 min. Coimmunoprecipitation (co‐IP) assay and western blot analysis were performed as described previously [13].

### 2.6 Glutathione S‐transferase pull‐down assay

Cells were lysed with the RIPA buffer (P0013B, Beyotime Biotechnology) on ice for 30 min. Glutathione S‐transferase (GST) tagged protein were absorbed by GST beads (P2132, Beyotime Biotechnology) and co‐cultured with cell lysates for 6 h. The GST beads were boiled for 10 min and subjected to western blot analysis.

### 2.7 Annexin V/PE FACS analysis

Cells with distinct treatment were washed with PBS three times. 1 × 10000 cells were suspended with PBS and subjected to Annexin V/PE analysis following the instruction of Annexin V‐PE/ 7‐AAD cell apoptosis detection kit (P‐CA‐206, Procell, Wuhan, China).

### 2.8 Subcellular fractionation and PARP trapping assay

The nuclear and cytoplasmic proteins were extracted by using the Nuclear and Cytoplasmic Protein Extraction Kit (P0028, Beyotime Biotechnology). Briefly, cells were lysed by added 200 µL of PMSF cytoplasmic protein extraction reagent A on ice for 15 min. Then, 10 µL of Cytoplasmic Protein Extraction Reagent B was added to the cells for 1 min. Cells were centrifuged 12 000–16 000 ***g*** for 5 min, and the supernatant was the cytoplasmic protein. 50 µL of nucleoprotein extraction reagent was added to the precipitate, and the supernatant was collected for nucleoprotein after 30 min on ice and centrifugation at 12 000–16 000 ***g*** for 10 min.

For PARP trapping assay, collected the precipitate after nucleoprotein extraction reagent treatment, washed the precipitate three times with nucleoprotein extraction reagent, and then digested with 5 units of micrococcal nuclease to release chromatin‐bound proteins. PARP binding in the chromatin was detected by western blot analysis.

### 2.9 Statistical analysis

The experimental data are presented as mean ± standard deviation (mean ± SD). The sample size (*n*) for each statistical analysis is provided in the figure legends. graphpad prism 5 software (San Diego, CA, USA) was used to calculate the *P* value using the unpaired two‐sided Student’s *t*‐test for comparison of difference between two groups, or one‐way ANOVA followed by Turkey’s multiple comparisons *post hoc* test for comparison of differences between more than two groups. Differences were considered statistically significant at *P* values <0.05. In all cases, statistical differences were considered at *P < 0.05; **P < 0.01; ***P < 0.001; not significant (ns), *P* > 0.05.

## 3 Results

### 3.1 FBP1 modulates PARP inhibitor sensitivity in pancreatic cancer cells

**Fig. 1:**
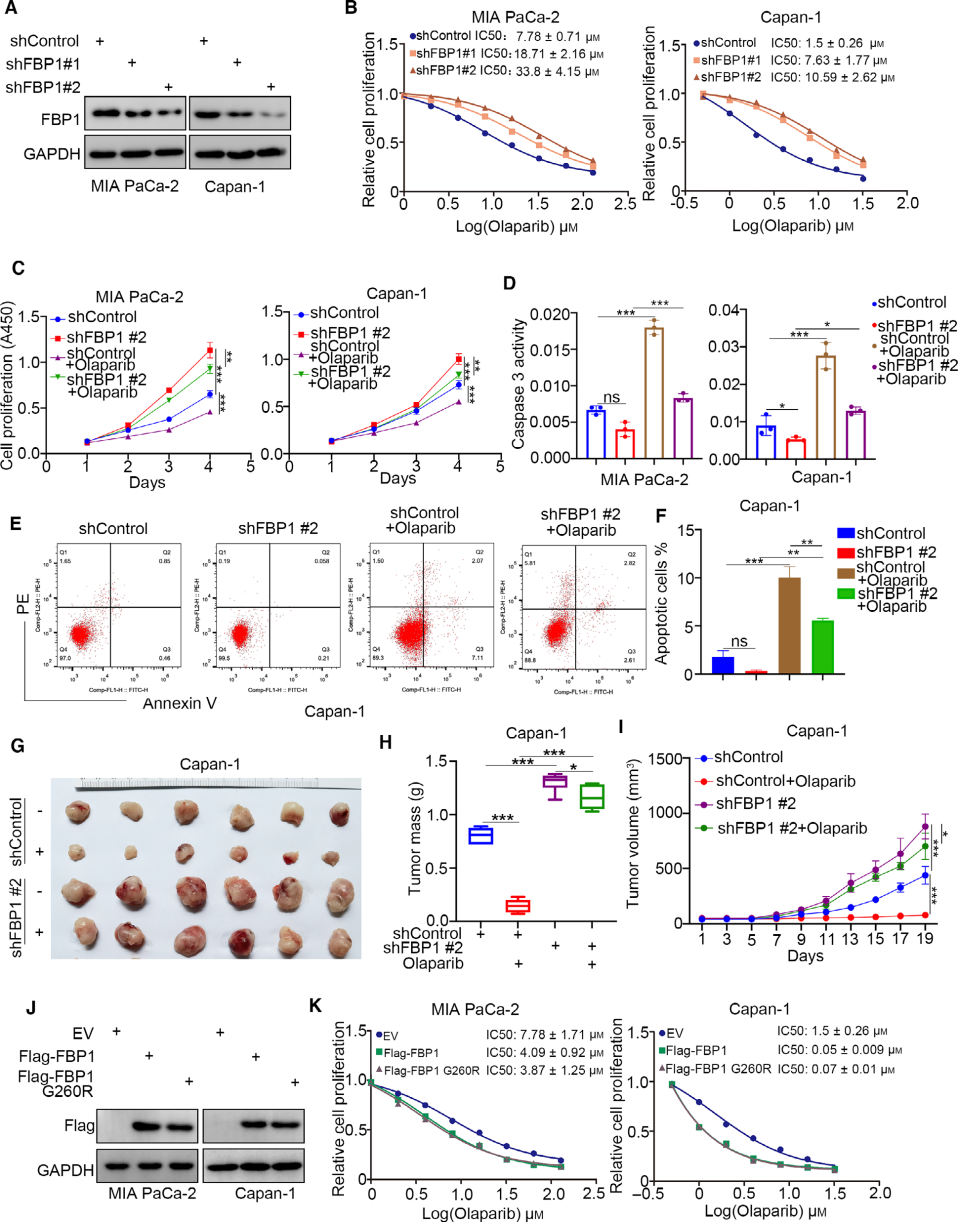
FBP1 modulates PARP inhibitor sensitivity in pancreatic cancer cells. (A and B) MIA PaCa‐2 and Capan‐1 cells were infected with indicated shRNAs for 72 h. Cells were harvested for western blot analysis (A) and treated with a serial concentration of Olaparib for measuring the IC50 values of Olaparib (B), which repeated for three replicates. (C–F) MIA PaCa‐2 and Capan‐1 cells were infected with indicated shRNAs for 72 h. Cells were harvested for CCK‐8 assay (C), caspase 3 activity assay (D), and Annexin V/PE facs assay (E and F). Data are shown as mean ± SD (*n* = 3). Statistical analyses were performed with one‐way ANOVA followed by Tukey's multiple comparisons tests. ns, not significant; *P < 0.05; **P < 0.01; ***P < 0.001. (G–I) Capan‐1 cells were infected with indicated shRNAs for 72 h. After the puromycin selection, cells were subcutaneously injected into the nude mice. The mice were treated with or without Olaparib (10 mg·kg^−1^·day^−1^, i.p., 2 weeks). Representative tumor images (G), tumor weights (H), and tumor growth curves (I) are shown. Data are shown as mean ± SD (*n* = 6). Statistical analyses were performed with one‐way ANOVA followed by Tukey's multiple comparisons tests. *P < 0.05; ***P < 0.001. (J and K) MIA PaCa‐2 and Capan‐1 cells were transfected with indicated plasmids for 48 h. Cells were harvested for western blot analysis (J) and treated with a serial concentration of Olaparib for measuring the IC50 values of Olaparib (K), which repeated for three replicates.

We have previously reported that FBP1 regulates the sensitivity of pancreatic cancer to various types of small molecules, including BET inhibitors, gemcitabine, MEK inhibitors, and AKT inhibitors [13, 14, 15]. PARP inhibitors (such as Olaparib) are newly identified small molecules [19], and we aimed to investigate whether FBP1 contributed to the response of pancreatic cancer cells to this agent. First, we chose MIA PaCa‐2 (BRCA1/2 wild type) and Capan‐1 (BRCA2 mutant type) cells for the subsequent study [16]. FBP1‐specific short hairpin RNAs (shRNAs) were transfected into pancreatic cancer cells to construct FBP1 stable knockdown cells after puromycin selection (Fig. 1A). We found that silencing FBP1 increased the half‐maximal inhibitory concentration (IC50) values of Olaparib in both MIA PaCa‐2 and Capan‐1 cells (Fig. 1B). Consistent with the previous study, we also showed that knockdown of FBP1 decreased the sensitivity of MIA PaCa‐2 and Capan‐1 cells to gemcitabine, MEK inhibitors, and AKT inhibitors (Fig. S1A–F). Moreover, CCK‐8, caspase 3 activity, and Annexin V/PE assays demonstrated that FBP1 knockdown resulted in pancreatic cancer cell resistance to Olaparib *in vitro* (Fig. 1C–F). Unsurprisingly, knockdown of FBP1 decreased the sensitivity to Olaparib in the subcutaneous xenograft model generated by using Capan‐1 cells (Fig. 1G–I). In contrast, when WT FBP1 and G260R enzymatically dead mutants were transfected into MIA PaCa‐2 and Capan‐1 cells, overexpression of FBP1 sensitized the pancreatic cancer cells to Olaparib, which was independent of its glucogenic activity (Fig. 1J,K). Thus, our results indicated that FBP1 regulates the sensitivity of pancreatic cancer cells to Olaparib. Notably, knockdown or overexpression of FBP1 not only changed the sensitivity of Capan‐1 with BRCA2 mutant type to Olaparib, but also affected MIA PaCa‐2 cells with WT BRCA1/2. The BRCA2 mutation status seems to be dispensable for the effect of FBP1 on the sensitivity of pancreatic cancer to Olaparib. However, the BRCA2 alterations are not the only alterations responsible for homologous recombination (HR) defects and Olaparib sensitivity [20]. The specific mechanism by which FBP1 regulates the sensitivity of pancreatic cancer to Olaparib and whether FBP1 can modulate the sensitivity to Olaparib without affecting the HR pathway needs further study.

### 3.2 FBP1 interacts with DNMT1 in pancreatic cancer cells

**Fig. 2:**
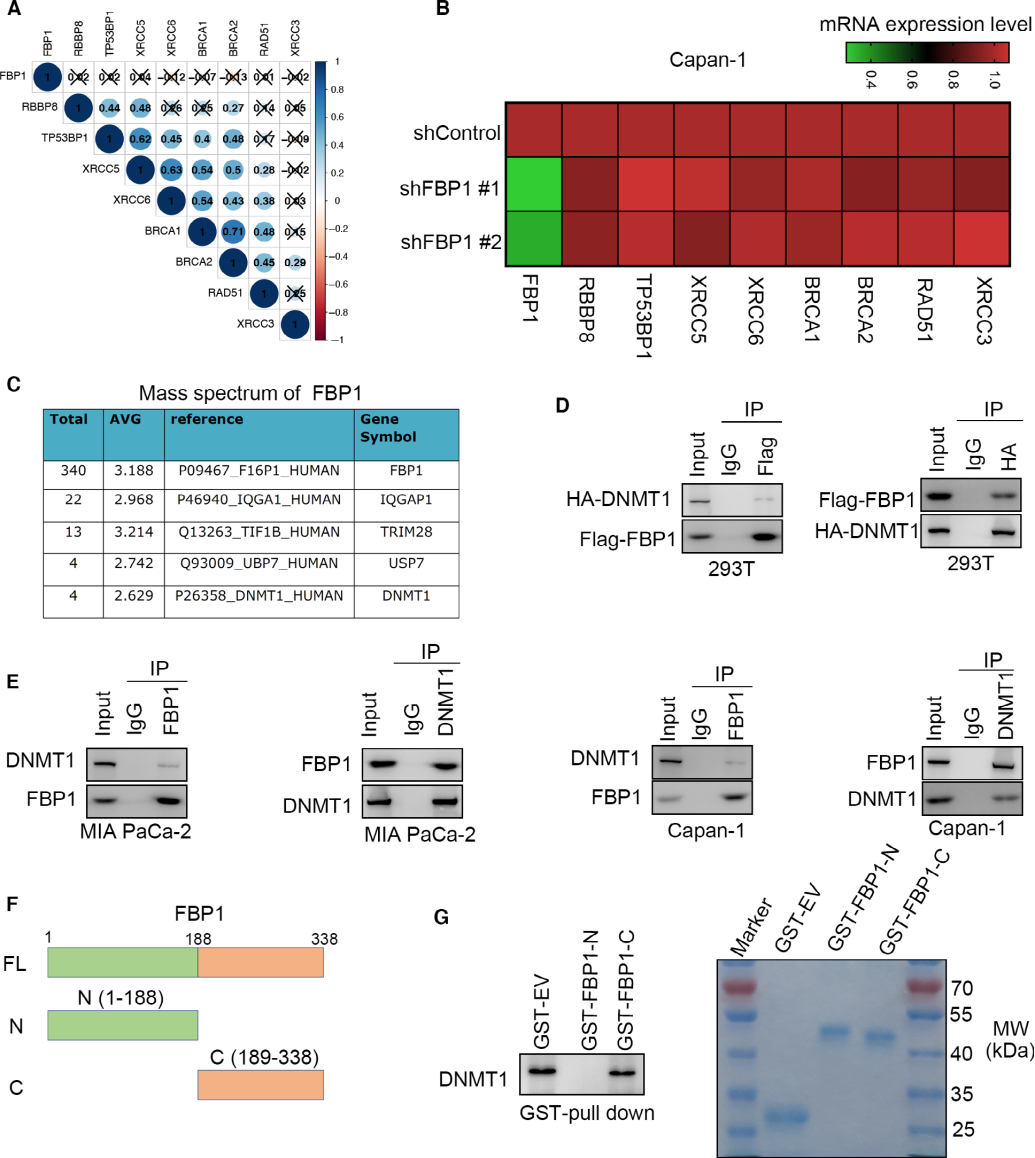
FBP1 interacts with DNMT1 in pancreatic cancer cells. (A) Gene expression correlation between FBP1 and DNA repair associated genes was performed by Pearson correlation analysis, which was analyzed for three times. (B) Capan‐1 cells were infected with indicated shRNAs for 72 h. Cells were collected for qRT‐PCR analysis. Heatmap showed the expression level of genes. This experiment was repeated for three replicates. (C) the mass spectrometry of FBP1 in 293T cells was shown. (*n* = 1 for IgG and FBP‐1 group) (D) 293T cells were transfected indicated plasmids for 48 h. Cell were harvested for co‐IP assay by using the Flag and HA antibodies, respectively, which repeated for three replicates. (E) MIA PaCa‐2 and Capan‐1 cells were collected for co‐IP assay by using the FBP1 and DNMT1 antibodies, respectively, which repeated for three replicates. (F) A schematic diagram depicts the domain of FBP1. (G) Western blotting analysis of DNMT1 GST‐pulled down by FBP1 recombinant, which repeated for three replicates. The marker for western blot was used to indicate the specific size of molecular weight of GST‐recombinant proteins.

Based on the above findings, we investigated the underlying mechanism of how FBP1 contributes to sensitivity to PARP inhibitors in pancreatic cancer. We analyzed the TCGA dataset and found that there was no correlation between FBP1 and other genes associated with DNA DSBs [21] (Fig. 2A). This result was also confirmed in Capan‐1 cells (Fig. 2B). Then, we analyzed the mass spectrometry data of FBP1 reported previously [22]. The mass spectrometry data identified several binding partners of FBP1, such as IQGAP1, TRIM28, USP7, and DNMT1 (Fig. 2C). We previously showed that FBP1 could interact with IQGAP1 to inhibit the activation of the MAPK pathway and that TRIM28 bound to FBP1 to promote FBP1 degradation [13, 22]. To identify new binding partners of FBP1 that might contribute to modulating the sensitivity to PARP inhibitors, we checked the unexplored candidates from the mass spectrometry of FBP1. It has been reported that DNMT1 interacts with PARP1 to regulate the sensitivity of breast cancer and acute myeloid leukemia cells to PARP inhibitors [11]. Thus, we aimed to study whether FBP1 regulated the sensitivity to Olaparib through DNMT1. First, co‐IP showed that exogenously expressed FBP1 reciprocally interacted with DNMT1 in 293T cells (Fig. 2D). Then, we demonstrated that endogenous FBP1 bound to DNMT1 in both MIA PaCa‐2 and Capan‐1 cells (Fig. 2E). In addition, we divided the FBP1 protein into the N terminus and C terminus, as shown in Fig. 2F. GST pulldown assays indicated that DNMT1 interacted with the full‐length (FL) and C‐terminal (C) recombinant protein of FBP1 *in vitro*. Therefore, our data suggested that DNMT1 was the binding partner of FBP1 in pancreatic cancer cells.

### 3.3 FBP1 determines the sensitivity to DNMT1‐dependent PARP inhibitors

**Fig. 3:**
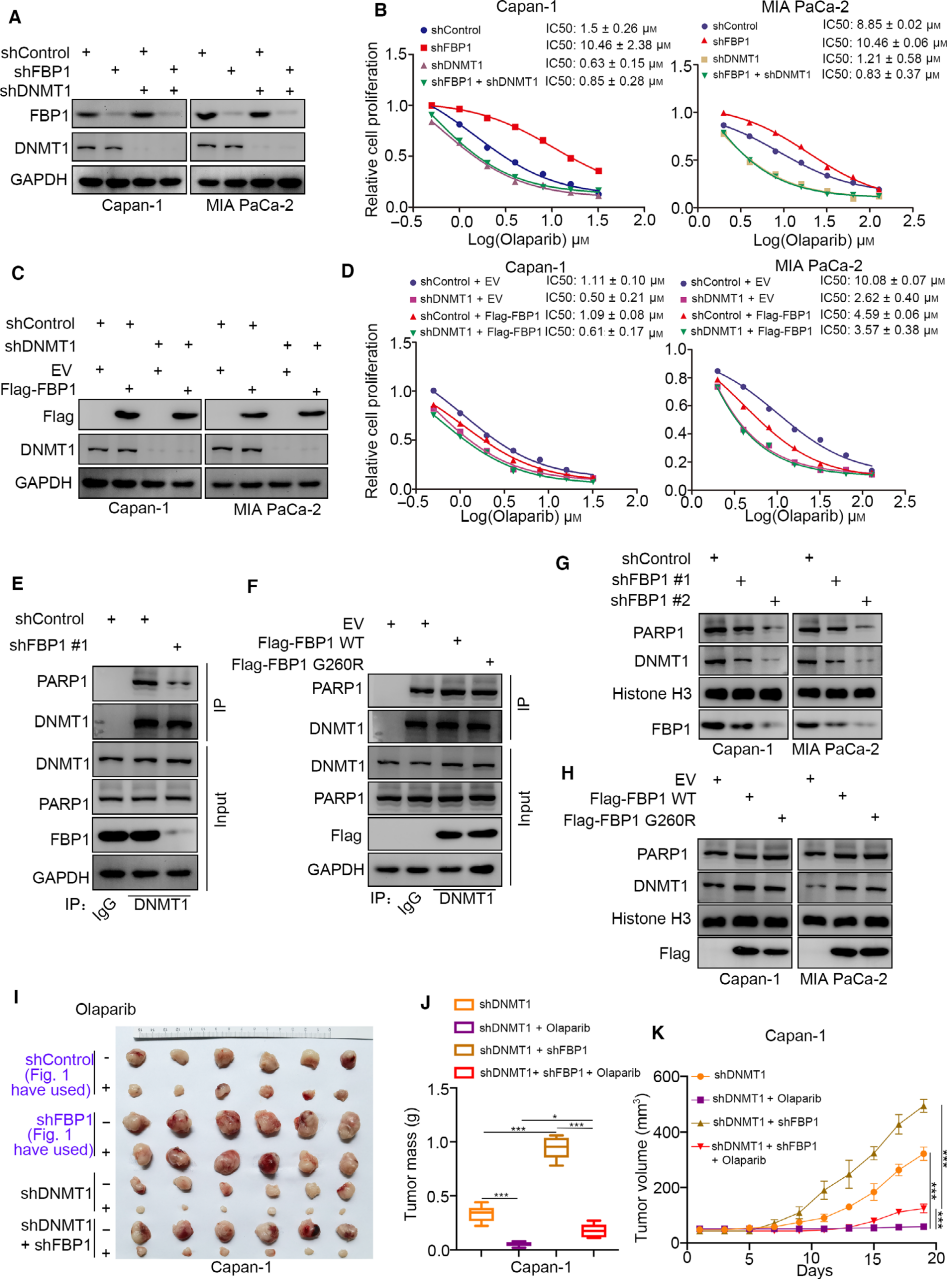
FBP1 determines the sensitivity to DNMT1‐dependent PARP inhibitors. (A and B) MIA PaCa‐2 and Capan‐1 cells were infected with indicated shRNAs for 72 h. Cells were harvested for western blot analysis (A) and treated with a serial concentration of Olaparib for measuring the IC50 values of Olaparib (B), which repeated for three replicates. (C and D) MIA PaCa‐2 and Capan‐1 cells were infected with indicated shRNAs for 48 h. Then, cells were transfected with empty vector or Flag‐FBP1 for another 24 h. Cells were harvested for western blot analysis (C) and treated with a serial concentration of Olaparib for measuring the IC50 values of Olaparib (D), which repeated for three replicates. (E) Capan‐1 cells were infected with indicated shRNAs for 72 h. Cells were harvested and underwent co‐IP assay by using the DNMT1 antibodies, which repeated for three replicates. (F) Capan‐1 cells were transfected with indicated plasmids for 48 h. Cells were harvested and underwent co‐IP assay by using the DNMT1 antibodies, which repeated for three replicates. (G) Capan‐1 cells were infected with indicated shRNAs for 72 h. Cells were harvested and underwent PARP trapping assay, which repeated for three replicates. (H) Capan‐1 cells were transfected with indicated plasmids for 48 h. Cells were harvested and underwent PARP trapping assay, which repeated for three replicates. (I–K) Capan‐1 cells were infected with indicated shRNAs for 72 h. After the puromycin selection, cells were subcutaneously injected into the nude mice. The mice were treated with or without Olaparib (10 mg·kg^−1^·day^−1^, i.p., 2 weeks). Representative tumor images (I), tumor weights (J), and tumor growth curves (K) are shown. Data are shown as mean ± SD (*n* = 6). Statistical analyses were performed with one‐way ANOVA followed by Tukey's multiple comparisons tests. *P < 0.05; ***P < 0.001. The ruler on the top of the representative tumor images on the panel I was used to indicate the specific size of tumors.

Since DNMT1 is responsible for the resistance of cancer cells to PARP inhibitors, we investigated whether FBP1 regulates the sensitivity to PARP inhibitors via DNMT1. First, we performed FBP1 silencing or overexpression after knockdown of DNMT1 in Capan‐1 and MIA PaCa‐2 cells, which were subjected to western blot analysis (Fig. 3A,C) or MTS assay by treatment with a serial dose of Olaparib (Fig. 3B,D). Notably, DNMT1 knockdown diminished the change in the IC50 values of Olaparib after FBP1 silencing or overexpression in both Capan‐1 and MIA PaCa‐2 cells (Fig. 3A–D). These data suggested that DNMT1 might be the key mediator of FBP1‐induced changes in the sensitivity of pancreatic cancer cells to PARP inhibitors. In addition, we observed that knockdown or overexpression of FBP1 had no effect on the protein level of DNMT1 (Fig. 3A,C), which suggests that other effectors might cooperate with FBP1 or DNMT1 to regulate the sensitivity to PARP inhibitors. It has been reported that DNMT1 interacts with PARP1 to form a complex, and DNMT1 inhibitors were found to promote the DNMT1 and PARP1 complex trapped in chromatin [11], which is the molecular basis for DNMT1 inhibitors to enhance the antitumor effect of PARP inhibitors [11]. Our study also revealed that PARP1 depletion attenuated the changes in PARP inhibitors sensitivity after knockdown or overexpression of FBP1 in pancreatic cancer cells (Fig. S2A–D). Thus, we hypothesized that FBP1 might influence the DNMT1‐PARP1 complex in cells. Interestingly, we showed that knockdown of FBP1 decreased the interaction between DNMT1 and PARP1 in Capan‐1 cells (Fig. 3E). Overexpression of FBP1 WT and the FBP1 G260R mutant resulted in increased binding of PARP1 and DNMT1 in Capan‐1 cells (Fig. 3F). Then, we investigated whether FBP1 influenced DNMT1 and PARP1 complex binding to chromatin. Notably, we showed that FBP1 silencing attenuated DNMT1 and PARP1 binding to chromatin in Capan‐1 and MIA Paca‐2 cells (Fig. 3G). In contrast, overexpression of WT FBP1 and the G260R mutant trapped more DNMT1 and PARP1 in the chromatin (Fig. 3H). Furthermore, we showed that knockdown of DNMT1 enhanced the sensitivity to Olaparib in vivo, and co‐knockdown of FBP1 and DNMT1 could not reverse the effect on Olaparib resistance induced by knockdown of FBP1 alone (Fig. 3I–K). In addition, we detected the IC50 values of DNMT inhibitors (decitabine) in MIA PaCa‐2 and Capan‐1 cells after knockdown or overexpression of FBP1. We showed that FBP1 silencing increased the IC50 values of decitabine in MIA PaCa‐2 and Capan‐1 cells (Fig. S2E,F). However, overexpression of FBP1 resulted in a decrease in the IC50 values of decitabine in MIA PaCa‐2 and Capan‐1 cells (Fig. 2G,H). Together, these data indicated that FBP1 contributed to sensitivity to PARP1 inhibitors through the DNMT1‐PARP1 complex in pancreatic cancer.

### 3.4 The nuclear localization of FBP1 enhances the antitumor effect of PARP inhibitors in pancreatic cancer

**Fig. 4:**
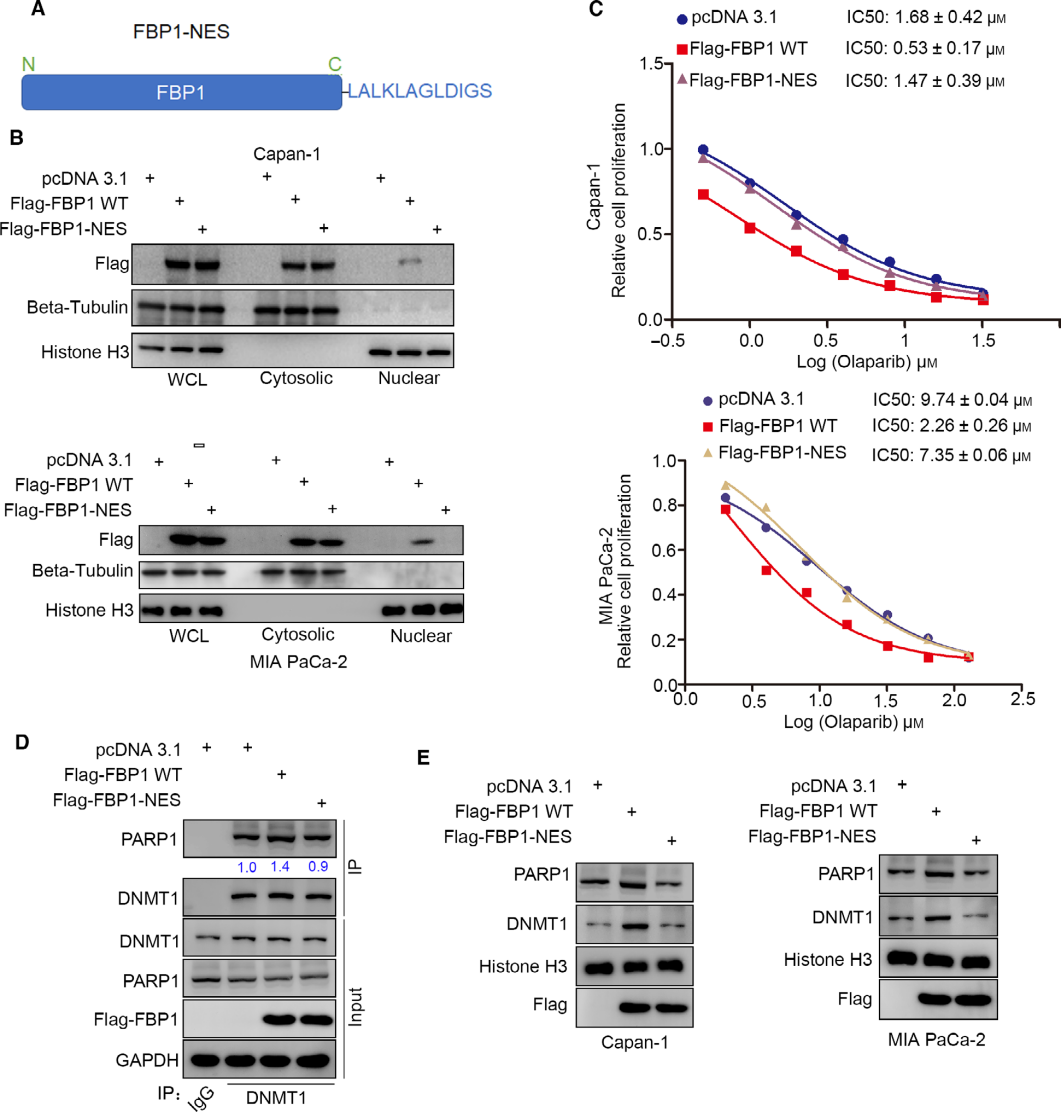
Nuclear localization of FBP1 enhances the antitumor effect of PARP inhibitors in pancreatic cancer. (A) a schematic diagram depicted the nuclear exporting sequence of FBP1. (B and C) MIA PaCa‐2 and Capan‐1 cells were transfected with indicated plasmids for 48 h. Cells were harvested for subcellular fractionation and western blot analysis (B) or treated with a serial concentration of Olaparib for measuring the IC50 values of Olaparib (C), which repeated for three replicates. (D) Capan‐1 cells were transfected with indicated plasmids for 48 h. Cells were harvested and underwent co‐IP assay by using the DNMT1 antibodies, which repeated for three replicates. (E) Capan‐1 cells were transfected with indicated plasmids for 48 h. Cells were harvested and underwent PARP trapping assay, which repeated for three replicates.

FBP1 is a rate‐limiting enzyme in gluconeogenesis and mainly inhibits cancer cell progression in the cytoplasm. Li et al. [23] showed that FBP1 bound to HIF protein in the nucleus to suppress renal and hepatocellular cancer progression. We next studied the distribution of FBP1 to determine whether it affects the sensitivity of cancer cells to PARP inhibitors. First, we constructed an FBP1 nucleus exporting (NES) mutant to prevent FBP1 localization in the nucleus [23] (Fig. 4A). Western blot analysis confirmed that the FBP1‐NES mutant was not translocated to the nucleus (Fig. 4B). The IC50 assay of Olaparib demonstrated that the FBP1‐NES mutant had no effect on the sensitivity to Olaparib compared with pcDNA3.1 in either Capan‐1 or MIA PaCa‐2 cells (Fig. 4C). Moreover, we showed that FBP1‐NES did not increase the binding of DNMT1 and PARP1 in Capan‐1 cells. Furthermore, WT FBP1 but not FBP1‐NES trapped the DNMT1 and PARP1 complex in chromatin in Capan‐1 and MIA PaCa‐2 cells (Fig. 4E). Thus, these data suggested that the subcellular distribution of FBP1 was essential for modulating the sensitivity of pancreatic cancer cells to PARP inhibitors.

### 3.5 USP7 binds with FBP1 and hinders FBP1 translocation to the nucleus in pancreatic cancer

**Fig. 5:**
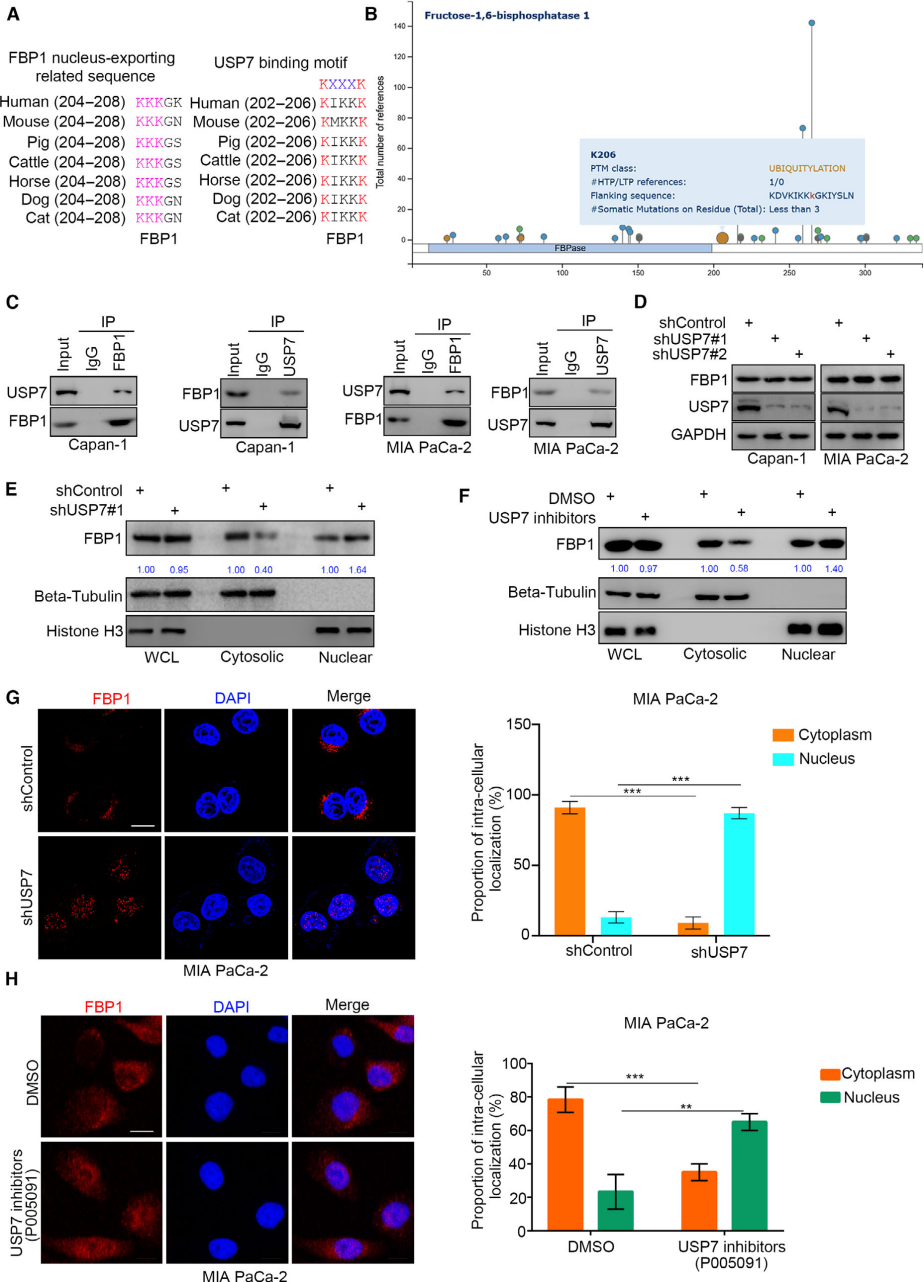
USP7 binds with FBP1 and hinders FBP1 translocation to the nucleus in pancreatic cancer. (A) a schematic diagram depicted the nuclear exporting related sequence and USP7 binding sequence of FBP1. (B) the ubiquination site of FBP1 indicated by PhosphoSitePlus dataset. (C) MIA PaCa‐2 and Capan‐1 cells were collected for co‐IP assay by using the FBP1 and USP7 antibodies, respectively, which repeated for three replicates. (D) MIA PaCa‐2 and Capan‐1 cells were infected with indicated shRNAs for 72 h. Cells were harvested for western blot analysis, which repeated for three replicates. (E) MIA PaCa‐2 cells were infected with indicated shRNAs for 72 h. Cells were subjected to subcellular fractionation and western blot analysis, which repeated for three replicates. F, MIA PaCa‐2 cells were treated with or without USP7 inhibitors (10 μm) for 24 h. Cells were subjected to subcellular fractionation and western blot analysis, which repeated for three replicates. (G) MIA PaCa‐2 cells were infected with indicated shRNAs for 72 h. Cells were subjected to immunofluorescence detection by using the FBP1 antibodies. The size of scale bar on microscopy image was 5μm. Data are shown as mean ± SD (*n* = 3). Statistical analyses were performed with one‐way ANOVA followed by Tukey's multiple comparisons tests. ***P < 0.001. (H) MIA PaCa‐2 cells were treated with or without USP7 inhibitors (10 μm) for 24 h. Cells were subjected to immunofluorescence detection by using the FBP1 antibodies. The size of scale bar on microscopy image was 5μm. Data are shown as mean ± SD (*n* = 3). Statistical analyses were performed with one‐way ANOVA followed by Tukey's multiple comparisons tests. **P < 0.01; ***P < 0.001.

Since the nuclear localization of FBP1 in pancreatic cancer is crucial for the sensitivity of pancreatic cancer to PARP inhibitors, the regulatory mechanism by which FBP1 is translocated to the nucleus needs to be further studied. The “^204^KKKGK^208^” sequence in the N‐terminal region of FBP1 determines the nuclear localization of FBP1 in cells [24]. Coincidentally, the USP7 binding motif partially overlapped with the “^204^KKKGK^208^” sequence (Fig. 5A). The bioinformatics analysis also showed that K206 of FBP1 undergoes ubiquitination modification (Fig. 5B). Moreover, the subsequent co‐IP assay demonstrated that USP7 interacted with FBP1 reciprocally in pancreatic cancer cells (Fig. 5C). Meanwhile, we constructed an FBP1 mutant with a USP7 binding motif deletion (Δ^202^KIKKKGK^208^; Fig. S3A). We showed that USP7 could not bind to the FBP1 Δ^202^KIKKKGK^208^ mutant (Fig. S3A). USP7 is a deubiquitinase and regulates the stability of target proteins i4n cells [25]. However, knockdown of USP7 did not change the protein level of FBP1 in Capan‐1 and MIA PaCa‐2 cells (Fig. 5D), which indicates that USP7 has no effect on the protein stabilization of FBP1. The USP7 binding motif partially overlapped with the “^204^KKKGK^208^” sequence of FBP1, which determined the nuclear localization of FBP1 in cells. The FBP1 Δ^202^KIKKKGK^208^ mutant showed increased cytoplasmic localization, as shown in Fig. S3B–D. Furthermore, we found that USP7 silencing induced FBP1 translocation to the nucleus (Fig. 5E,G). Similarly, treatment with USP7 inhibitors (10 μm, not the lethal dose for MIA PaCa‐2 as indicated in Fig. S3E) also increased the nuclear proportion of FBP1 in MIA PaCa‐2 cells (Fig. 5F,H). In contrast, overexpression of USP7 reduced the nuclear proportion of FBP1 in MIA PaCa‐2 cells (Fig. 3F,G). Thus, our results demonstrated that USP7 might play an important role in the subcellular localization of FBP1 in cells.

### 3.6 FBP1‐K206 ubiquitination increases the sensitivity of pancreatic cancer to PARP inhibitors

**Fig. 6:**
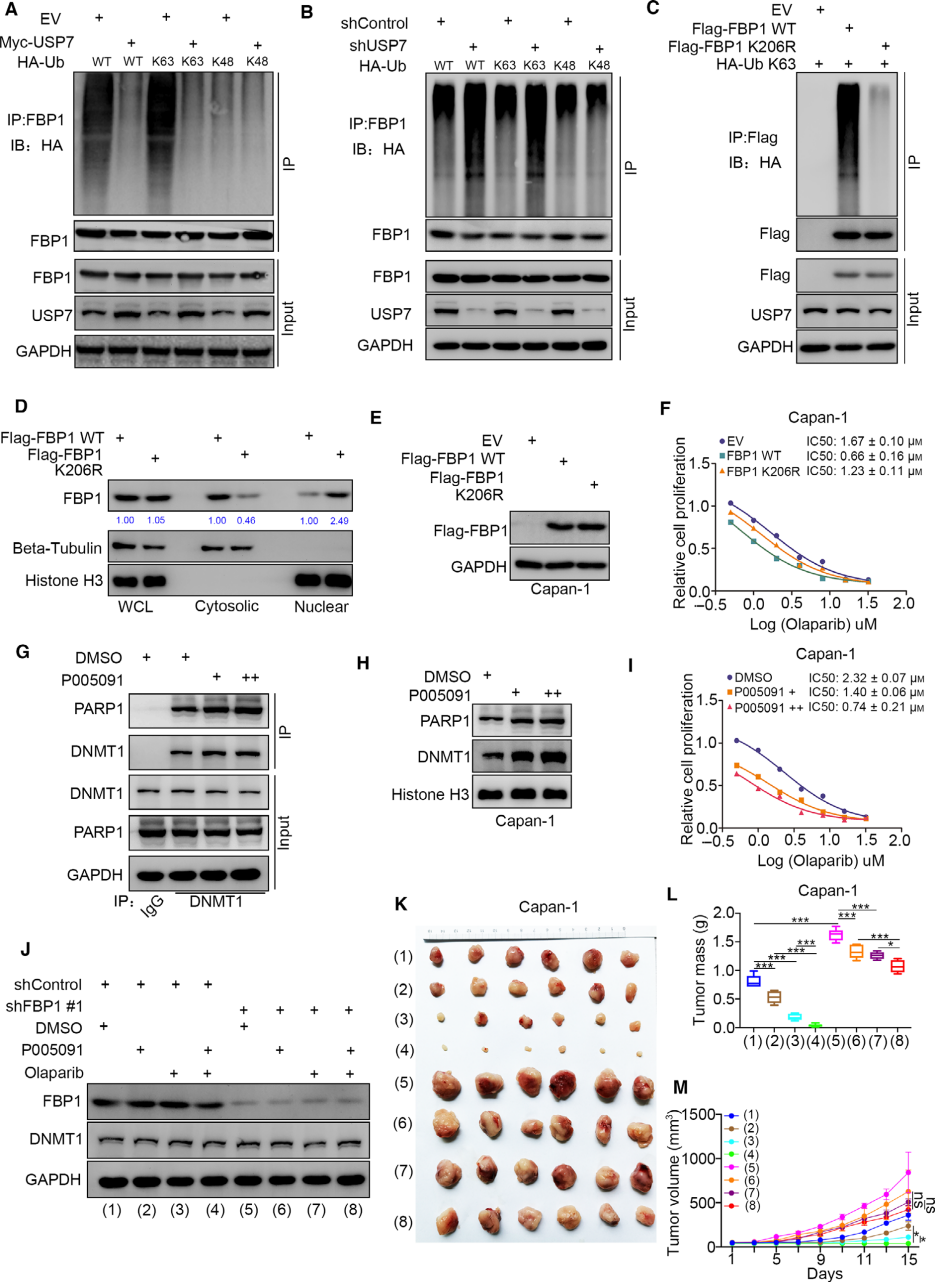
FBP1‐K206 ubiquitination increases the sensitivity of pancreatic cancer to PARP inhibitors. (A) Capan‐1 cells were transfected with indicated constructs for 48 h. Cells were treated with MG132 for 8 h and subjected to IP assay by using FBP1 antibodies, which repeated for three replicates. (B) Capan‐1 cells were infected with shControl or shUSP7 for 24 h. Then, these cells were transfected with indicated constructs for 48h. Cells were treated with MG132 for 8 h and subjected to IP assay by using FBP1 antibodies, which repeated for three replicates. (C) Capan‐1 cells were transfected with indicated constructs for 48 h. Cells were treated with MG132 for 8 h and subjected to IP assay by using FBP1 antibodies, which repeated for three replicates. (D) Capan‐1 were transfected with indicated plasmids for 48 h. Cells were harvested for subcellular fractionation and western blot analysis, which repeated for three replicates. (E and F) Capan‐1 cells were transfected with indicated plasmids for 48 h. Cells were harvested for western blot analysis (E) and treated with a serial concentration of Olaparib for measuring the IC50 values of Olaparib (F), which repeated for three replicates. (G–I) Capan‐1 cells were treated with or without USP7 inhibitors (10 μm) for 24 h. Cells were harvested for IP assay by using DNMT1 antibodies (G), PARP trapping assay (H), and treated with a serial concentration of Olaparib for measuring the IC50 values of Olaparib (I), which repeated for three replicates. (J–M) Capan‐1 cells were infected with indicated shRNAs for 72 h. After puromycin selection, cells were harvested for western blot analysis (J) and subcutaneously injected into the nude mice. The western blot analysis was repeated for three replicates. The mice were treated with or without Olaparib (10 mg·^−1^kg·day^−1^, i.p., 2 weeks) or USP7 inhibitors (10 mg·kg^−1^, i.p., twice a week). Representative tumor images (K), tumor weights (L), and tumor growth curves (M) are shown. Data are shown as mean ± SD (*n* = 6). Statistical analyses were performed with one‐way ANOVA followed by Tukey's multiple comparisons tests. ns, not significant; *P < 0.05; ***P < 0.001. The ruler on the top of the representative tumor images on panel K was used to indicate the specific size of tumors.

As USP7 is responsible for the subcellular distribution of FBP1 but does not influence the stability of FBP1 in pancreatic cancer cells, we were curious about how USP7 regulates this process. The ubiquitination assay showed that overexpression of USP7 specifically decreased K63‐linked ubiquitination of FBP1 in cells (Fig. 6A) and had no change on the K1‐, K11‐, K27‐, K29‐, K33‐, and K48‐linked ubiquitination of FBP1 (Figs 6A and S4A). In contrast, we also found that USP7 knockdown or USP7 inhibitor treatment promoted the K63‐linked ubiquitination of FBP1 (Figs 6B and S4B). Then, we demonstrated that the FBP1‐K206R mutant appreciably reduced the K63‐linked ubiquitination of FBP1 in Capan‐1 cells (Fig. 6C). Interestingly, we revealed that the FBP1‐K206R mutant promoted FBP1 translocation into the nucleus (Fig. 6D), which might be on account of a reduced interaction with USP7 in cells. Given that the nuclear localization of FBP1 is critical for modulating the sensitivity to the PARP inhibitors, as described above, we aimed to evaluate the effect of the FBP1‐K206R mutant on the response of pancreatic cancer to PARP inhibitors. We showed that overexpression of the FBP1‐K206R mutant increased the IC50 values of Olaparib compared with that of the WT FBP1 in both Capan‐1 and MIA PaCa‐2 cells (Figs 6F and S4C). Thus, these data indicated that K63‐linked ubiquitination at the FBP1‐K206 site is important for the translocation of FBP1 into the nucleus.

**Fig. 7:**
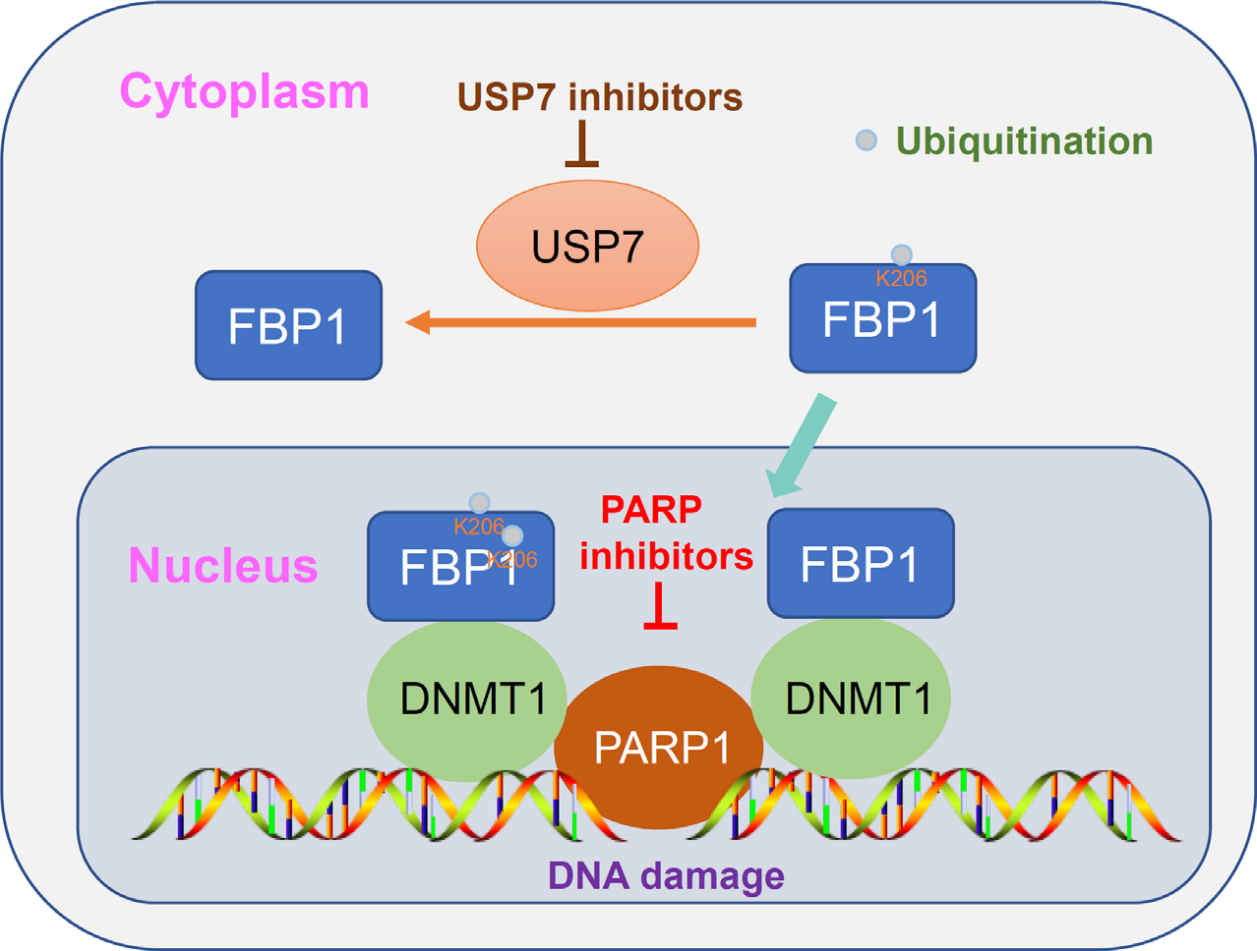
A model depicted that nuclear localized FBP1 interacted with DNMT1 and trapping PARP1 to the chromatin. USP7 bound with FBP1 and induced the deubiquitination modification of FBP1, which prevented FBP1 from translocation to nucleus. Thus, in combination with USP7 inhibitors and PARP inhibitors might manifest more powerfully antitumor effect than PARP inhibitors alone in pancreatic cancer cells.

USP7 inhibitors theoretically increase the ubiquitination of FBP1 by inhibiting USP7 activity. We showed that USP7 inhibitors enhanced the interaction between DNMT1 and PARP1 in cells (Fig. 6G). Consistent with previous findings, USP7 inhibitor treatment trapped more DNMT1 and PARP1 complexes in chromatin in MIA PaCa‐2 and Capan‐1 cells (Figs S4D and 6H). Furthermore, we demonstrated that USP7 inhibitors decreased the IC50 values of Olaparib in both Capan‐1 and MIA PaCa‐2 cells (Figs 6I and S4E). In addition, we showed that knockdown of USP7 or treatment with USP7 inhibitors could also slightly increase the interaction between DNMT1 and PARP1 and their localization to chromatin in Capan‐1 cells after FBP1 silencing (Fig. S4F–I), which might be partially due to the direct interaction and deubiquitination activity of USP7 on histone H3 and DNMT1 reported previously [26, 27]. Finally, we showed that USP7 inhibitors enhanced the antitumor effect of PARP inhibitors in mice, and this process was attenuated after knockdown of FBP1 (Fig. 6J–M). Therefore, our results indicated that the USP7/FBP1 axis modulated the sensitivity of pancreatic cancer to PARP inhibitors (Fig. 7).

## 4 Discussion

Since first‐line chemotherapeutic strategies (gemcitabine plus nab‐paclitaxel or fluorouracil‐based FOLFIRINOX) have shown limited effects in prolonging the lifespan of pancreatic cancer patients, Olaparib has been used in the clinic to treat pancreatic cancer patients with BRCA1/2 mutations [28]; these patients are sensitive to PARP inhibitors. The anticancer efficiency of PARP inhibitors is mainly determined by DNA damage repair gene defects through so‐called “synthetic lethality” [29]. Proteins associated with the restoration of HR repair, such as ATM, 53BP1, PALB2, and CHEK2, are closely correlated with the sensitivity to PARP inhibitors [30]. Thus, BRCA mutations are not the only mutations responsible for HR defects and Olaparib sensitivity [20]. PARP inhibitor monotherapy or combination therapy with other antitumor small molecules is in clinical trials for pancreatic ductal adenocarcinoma (PDAC) patients with BRCA mutations or mutations in other DNA damage repair genes [31].

Of note, developing drug resistance to PARP inhibitors has become a new problem for PDAC patients [32]. It has been reported that 40% of patients with BRCA germline mutations fail to respond to PARP inhibitors [33]. Therefore, studying the factors influencing the sensitivity to PARP inhibitors is essential for the survival of patients with pancreatic cancer. PARP inhibitors not only block the catalytic activity of PARP1 but also trap PARP1 on chromatin to stall replication forks [34]. PARP inhibitor‐resistant cells were shown to remodel the chromatin structure through an unknown mechanism [35]. This finding indicates that the ability of PARP1 to bind to chromatin also influences the antitumor effect of PARP inhibitors [36]. Similarly, DNMT inhibitors were proven to trap PARP1 in chromatin and enhance the antitumor efficiency of PARP inhibitors [11]. Here, we also found that FBP1 could trap PARP1 on chromatin, which might be one of the explanations for how FBP1 regulates the sensitivity of pancreatic cancer to PARP inhibitors.

FBP1 catalyzes the hydrolysis of fructose‐1,6‐bisphosphate to fructose 6‐phosphate in the presence of divalent cations and is recognized as the rate‐limiting enzyme of gluconeogenesis [37]. Due to the glucose metabolism‐related effect of FBP1, FBP1 functions as a tumor‐suppressing protein via agonist glycolysis in cancer cells [38, 39]. Interestingly, the anticancer effect of FBP1 is dispensable for metabolic activity [37]. In the cytoplasm, FBP1 was found to bind with IQGAP1 to inactivate the MAPK pathway and sensitize pancreatic cancer cells to gemcitabine [15]. In the nucleus, FBP1 was shown to directly interact with the HIF inhibitory domain to repress HIF activity in renal cancer and hepatocellular carcinoma [23]. In addition, FBP1 was found to inhibit the Wnt/β‐catenin pathway in cholangiocarcinoma cells [40]. Moreover, our previous study showed that FBP1 contributed to regulating the sensitivity of some anticancer agents. For instance, we showed that FBP1 interacted with BRD4 to enhance pancreatic cancer cell sensitivity to BET inhibitors [14]. Furthermore, we showed that FBP1 bound to STAT3 and prevented STAT3 phosphorylation, which modulated the immune response and PD‐L1 antibody blockade efficiency in pancreatic cancer cells [41]. Here, we showed that FBP1 regulated the sensitivity of pancreatic cancer cells to PARP inhibitors. However, the knockdown of FBP1 seemed to reduce the basal apoptosis in pancreatic cancer cells (Fig. 1), which might contribute to determining the sensitivity of PARP inhibitors. We also demonstrated that treatment with the PARP inhibitors after FBP1 knockdown resulted in more apoptosis than that in the control group, as shown in Fig. 1D,E, which indicated that reduced basal apoptosis after FBP1 silencing was not the only one reason FBP1 affected sensitivity to PARP inhibitors. Moreover, a subsequent study revealed that nuclear FBP1 interacted with DNMT1 to sensitize pancreatic cancer cells to PARP inhibitors, and this effect was independent of the enzymatic activity of FBP1. Then, we revealed that USP7‐mediated FBP1‐K206 ubiquitination played a key role in determining the nuclear translocation of FBP1. Further study showed that depletion of USP7 or inhibition of USP7 activity promoted FBP1 translocation to the nucleus and increased the interaction of PARP1 and DNMT1 and their localization to chromatin. Interestingly, we also found that FBP1 silencing decreased the interaction of PARP1 and DNMT1 and their localization to chromatin but did not completely abrogate the effect of USP7 on the interaction of PARP1 and DNMT1 and their localization to chromatin. USP7 deubiquitinates histone H3 to decrease the binding of DNMT1 to DNA methylation sites [26, 27]. Thus, FBP1 might be another mediator to explain the connection between USP7 and DNMT1 in cells.

Ubiquitination is an important post‐translation modification of FBP1. Our previous studies indicated that TRIM28‐dependent ubiquitination and USP44‐dependent deubiquitination of FBP1 regulated its stability in cancer cells [15, 22]. Unlike USP7‐mediated ubiquitination of FBP1 on lysine 206, we could not identify the specific lysine sites of FBP1 that were responsible for the ubiquitination or deubiquitination mediated by TRIM28 or USP44. Intriguingly, we noticed that TRIM28 and USP44 bound to the N terminus of FBP1, but the USP7 binding motif was located in the C‐terminal domain of FBP1. Thus, the regulatory mechanism of FBP1 is very complicated and needs to be studied further.

## 5 Conclusions

Collectively, we demonstrate that FBP1 regulates the sensitivity of pancreatic cancer to PARP inhibitors. Then, we showed that nuclear FBP1 is responsible for this process by interacting with DNMT1 and trapping PARP1 in chromatin. Moreover, we revealed that USP7 bound to FBP1 and induced the deubiquitination of FBP1, which prevented FBP1 from translocating to the nucleus (Fig. 7). Thus, USP7 inhibitors and PARP inhibitors might have more powerful antitumor effects than PARP inhibitors alone in PDAC patients.

## Conflict of interest

The authors declare no conflict of interest.

## Author contributions

XJ involved in methodology, writing—original draft, project administration; HW contributed to project administration; FG contributed to investigation and project administration; BZ contributed to conceptualization, formal analysis, and methodology; XC contributed to methodology, investigation, and project administration.

## Consent for publication

All subjects have written informed consent.

### Peer Review

The peer review history for this article is available at https://publons.com/publon/10.1002/1878‐0261.13149.

## Supporting information


**Fig. S1**. The IC50 values of Gemcitabine, MK2206, and JQ1 in both MIA PaCa‐2 and Capan‐1 cells after knockdown of FBP1.
**Fig. S2**. FBP1 regulates the sensitivity of PARP and DNMT1 inhibitors.
**Fig. S3**. USP7 inhibits the nuclear translocation of FBP1.
**Fig. S4**. The deubiquitination mediated by USP7 modulated the sensitivity of PARP inhibitors through the DNMT1/PARP1 complex.
**Table S1**. Sequences for primers used for shRNAs and RT‐qPCR.

## Data Availability

The datasets used and/or analyzed during the current study are available from the corresponding authors on reasonable request.
