# Supplementary material for: Deubiquitination of FBP1 by USP7 blocks FBP1–DNMT1 interaction and decreases the sensitivity of pancreatic cancer cells to PARP inhibitors
Source: Mol Oncol. 2021 Dec 17;16(7):1591–607. doi: 10.1002/1878-0261.13149 (PMC8978517; doi:10.1002/1878-0261.13149)
Supplement: Supplementary file 1 — Fig. S1. The IC50 values of Gemcitabine, MK2206, and JQ1 in both MIA PaCa‐2 and Capan‐1 cells after knockdown of FBP1. Fig. S2. FBP1 regulates the sensitivity of PARP and DNMT1 inhibitors. Fig. S3. USP7 inhibits the nuclear translocation of FBP1. Fig. S4. The deubiquitination mediated by USP7 modulated the sensitivity of PARP inhibitors through the DNMT1/PARP1 complex. Table S1. Sequences for primers used for shRNAs and RT‐qPCR. [file MOL2-16-1591-s001.docx]

**Supplementary Information**

**Deubiquitination of FBP1 by USP7 blocks FBP1–DNMT1 interaction and decreases the sensitivity of pancreatic cancer cells to PARP inhibitors**

Xiang Cheng, Bin Zhang, Feng Guo, Heshui Wu, Xin Jin


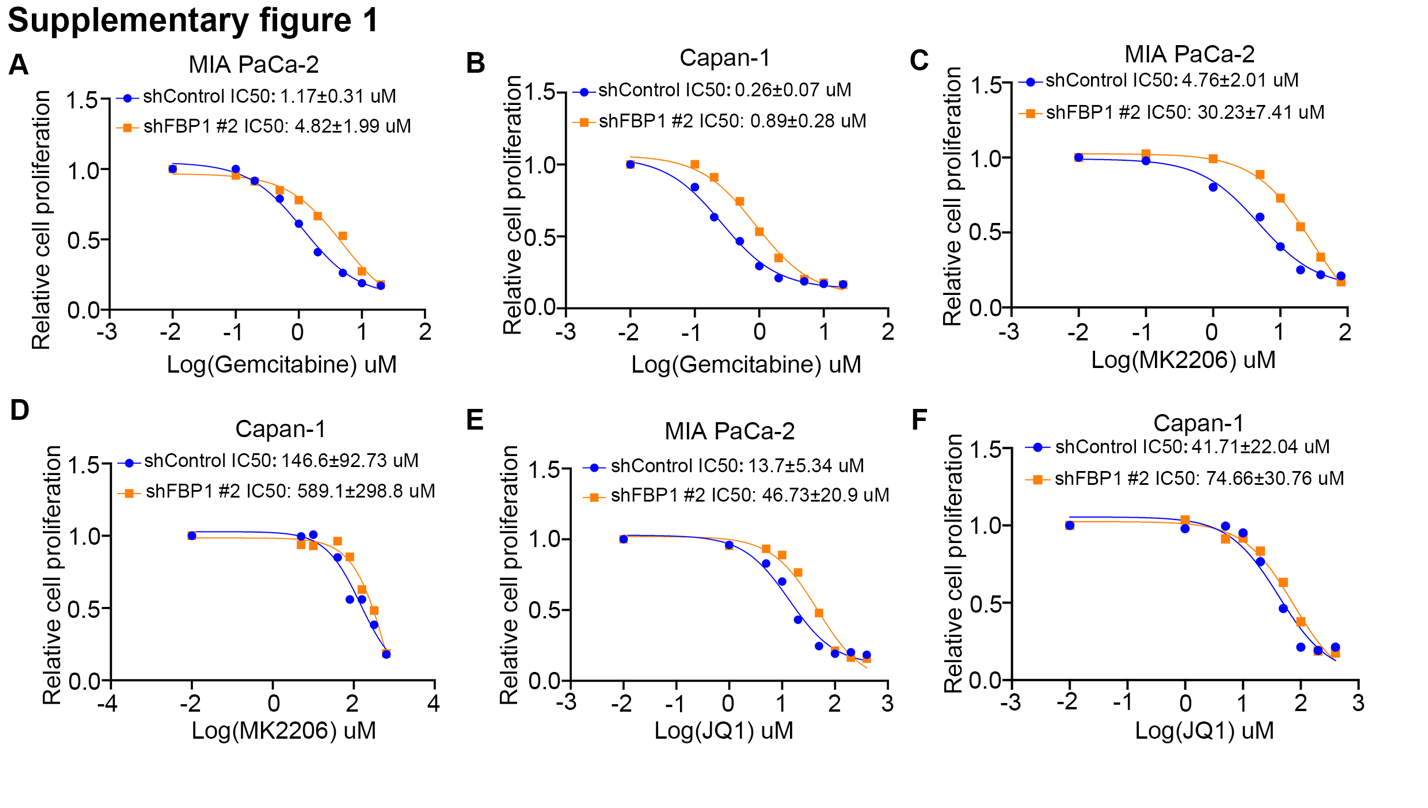


**Supplementary figure 1. The IC50 values of Gemcitabine, MK2206, and JQ1 in both MIA PaCa-2 and Capan-1 cells after knockdown of FBP1.**

MIA PaCa-2 and Capan-1 cells were infected with indicated shRNAs for 72 h. Cells were treated with a serial concentration of Gemcitabine, MK2206, and JQ1 for measuring the IC50 values of Gemcitabine (A and B), MK2206 (C and D), and JQ1 (E and F), which repeated for three replicates.

**
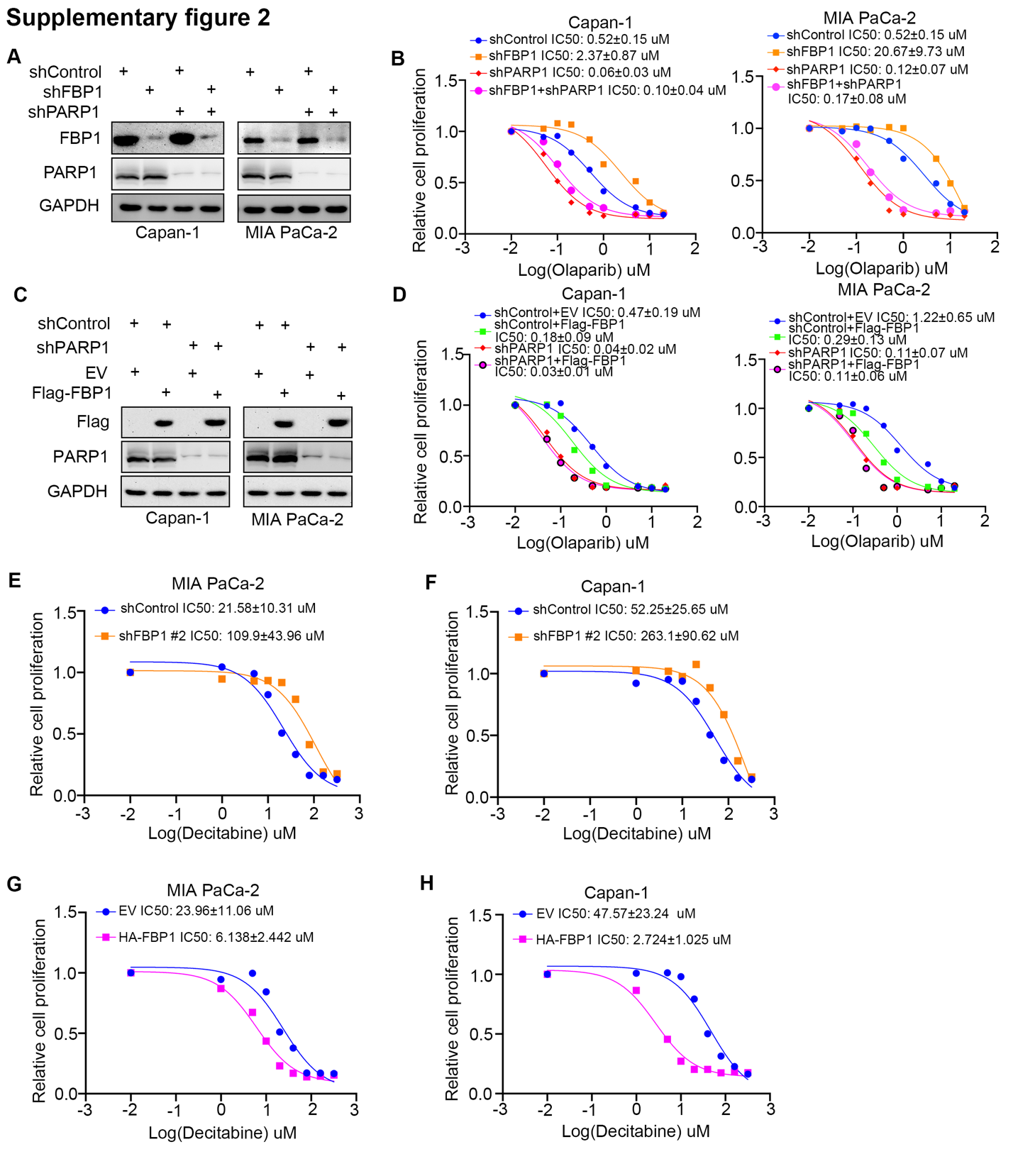
**

**Supplementary figure 2. FBP1 regulates the sensitivity of PARP and DNMT1 inhibitors.**

**A and B,** MIA PaCa-2 and Capan-1 cells were infected with indicated shRNAs for 72 h. Cells were harvested for western blot analysis (A) and treated with a serial concentration of Olaparib for measuring the IC50 values of Olaparib (B), which repeated for three replicates. **C and D**, MIA PaCa-2 and Capan-1 cells were infected with indicated shRNAs for 48 h. Then, cells were transfected with empty vector or Flag-FBP1 for another 24 h. Cells were harvested for Western blot analysis (C) and treated with a serial concentration of Olaparib for measuring the IC50 values of Olaparib (D), which repeated for three replicates. **E and F**, MIA PaCa-2 and Capan-1 cells were infected with indicated shRNAs for 72 h. Cells were treated with a serial concentration of Decitabine for measuring the IC50 values of Decitabine, which repeated for three replicates. **G and H**, MIA PaCa-2 and Capan-1 cells were transfected with indicated constructs for 48h. Cells were treated with a serial concentration of Decitabine for measuring the IC50 values of Decitabine, which repeated for three replicates.

**
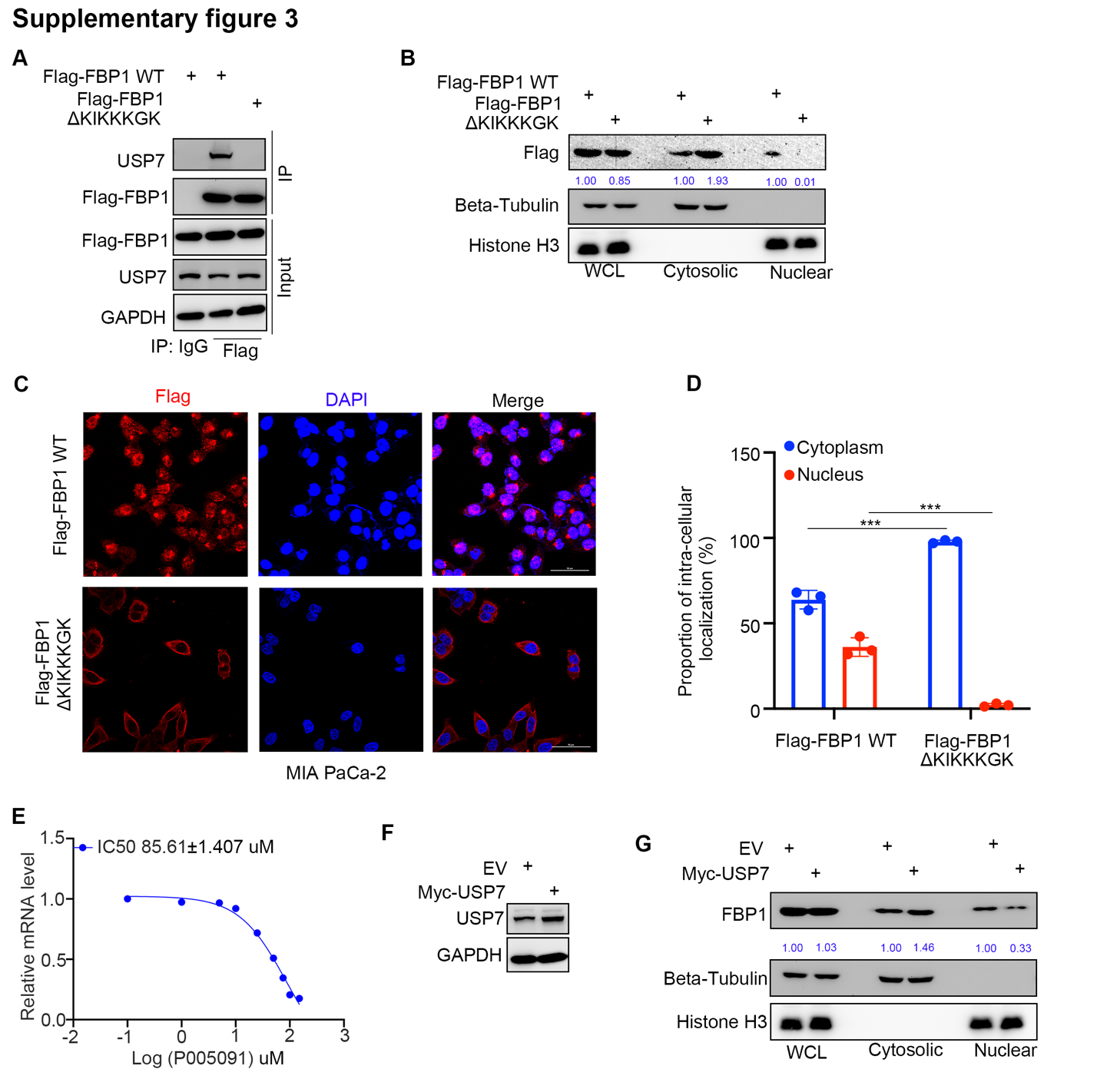
**

**Supplementary figure 3. USP7 inhibits the nuclear translocation of FBP1.**

**A**, MIA PaCa-2 cells were transfected with indicated plasmids for 48 h. Cells were harvested for IP assay by using the Flag-tagged antibody, which repeated for three replicates. **B**, MIA PaCa-2 cells were transfected with indicated plasmids for 48 h. Cells were subjected to subcellular fractionation and western blot analysis, which repeated for three replicates. **C and D**, MIA PaCa-2 cells were transfected with indicated plasmids for 48 h. Cells were subjected to immunofluorescence detection by using the FBP1 antibodies. Data are shown as mean ± SD (n = 3). Statistical analyses were performed with one-way ANOVA followed by Tukey's multiple comparison's tests. ***, P < 0.001. **E**, MIA PaCa-2 cells were treated with a serial concentration of P005091 for measuring the IC50 values, which repeated for three replicates. **F and G**, MIA PaCa-2 cells were transfected with indicated plasmids for 48 h. Cells were subjected to subcellular fractionation and western blot analysis, which repeated for three replicates.

**
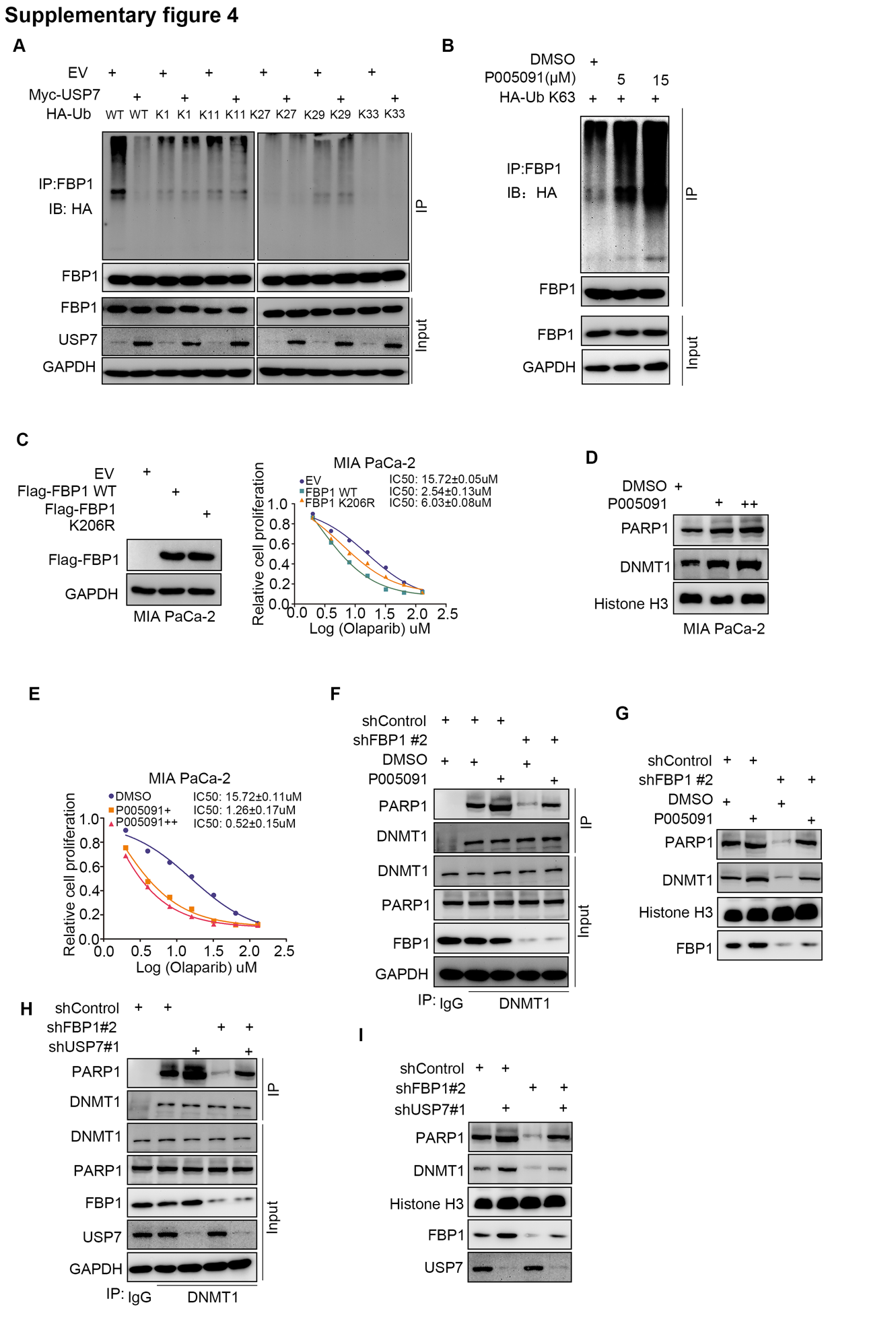
**

**Supplementary figure 4. The de-ubiquitination mediated by USP7 modulated the sensitivity of PARP inhibitors through the DNMT1/PARP1 complex.**

**A,** Capan-1 cells were transfected with indicated constructs for 48h. Cell were treated with MG132 for 8 h and subjected to IP assay by using FBP1 antibodies, which repeated for three replicates. **B**, Capan-1 cells were transfected with indicated constructs for 24h. Then, Capan-1 cells were treated with DMSO, P005091 (5 μM), and P005091 (15 μM) for another 24h. Cells were treated with MG132 for 8 h and subjected to IP assay by using FBP1 antibodies, which repeated for three replicates. **C,** MIA PaCa-2 cells were transfected with indicated plasmids for 48 h. Cells were harvested for western blot analysis and treated with a serial concentration of Olaparib for measuring the IC50 values of Olaparib, which repeated for three replicates. **D and E**, MIA PaCa-2 cells were treated with 10 μM (+) or 20 μM (++) USP7 inhibitors for 24 h. Cells were collected for PARP trapping assay (D) and treated with a serial concentration of Olaparib for measuring the IC50 values of Olaparib (E), which repeated for three replicates. **F and G**, MIA PaCa-2 cells were infected with indicated shRNAs for 24h. Then, MIA PaCa-2 cells were treated with 10μM (+) or 20μM (++) USP7 inhibitors for another 24 h. Cells were collected for IP assay by using the DNMT1 antibody (F) or PARP trapping assay (G), which repeated for three replicates. **H and I**, MIA PaCa-2 cells were infected with indicated shRNAs for 72h. Cells were collected for IP assay by using the DNMT1 antibody (H) or PARP trapping assay (I), which repeated for three replicates.

**Supplementary Table S1.** Sequences for primers used for shRNAs and RT-qPCR.

**Sequences for shRNAs**

| shFBP-1-1 | 5′-CCGGCCTTGATGGATCTTCCAACATCTCGAGATGTTGGAAGATCCATCAAGGTTTTTG-3′ |
| --- | --- |
| shFBP-1-2 | 5′-CCGGCGACCTGGTTATGAACATGTTCTCGAGAACATGTTCATAACCAGGTCGTTTTTG-3′ |
| shUSP7 | 5’-CCGGGACCAGCAATGTTAGATAACTCGAGTTATCTAACATTGCTGGTCTTTTT-3’ |
| shPARP1 | 5’-CCGGGGACCAAGTGTATGGTCAACTCGAGTTGACCATACACTTGGTCCTTTTT-3’ |
| shDNMT1 | 5’-CCGGGAAGAAGCACAGAAGTCAACTCGAGTTGACTTCTGTGCTTCTTCTTTTT-3’ |

**Sequences for primers used for RT-qPCR**

| **Species** | **Gene** | **Forward (5’-3’)** | **Reverse (5’-3’)** |
| --- | --- | --- | --- |
| Human | *GAPDH* | CCAGAACATCATCCCTGCCT | CCTGCTTCACCACCTTCTTG |
| Human | *FBP1* | TCAACTGCTTCATGCTGGAC | CGTAGACCAGAGTGCGATGA |
| Human | *RBBP8* | GCAGACAGTTTCTCCCAAGC | TGCCCAAGCAGTTTTCTTCT |
| Human | *TP53BP1* | TCTGTGAAGCAGCACCATTC | TTCTCTCCCCAACAATGAGG |
| Human | *XRCC5* | CCCCAATTCAGCAGCATATT | CCTTCAGCCAGACTGGAGAC |
| Human | *XRCC6* | AAAAGACTGGGCTCCTTGGT | TGTGGGTCTTCAGCTCCTCT |
| Human | *BRAC1* | GGTGGTACATGCACAGTTGC | ACTCTGGGGCTCTGTCTTCA |
| Human | *BRAC2* | AGCTCTTCACCCTGCAAAAA | CCAATGCCTCGTAACAACCT |
| Human | *RAD51* | CTCAGCCTCCCGAGTAGTTG | CATCACTGCCAGAGAGACCA |
| Human | *XRCC3* | AAGAAGGTCCCCGTACTGCT | GCCTCTGTCACCTGGTTGAT |
